# Is It Useful to Question the Recovery Behaviour of Patients with ME/CFS or Long COVID?

**DOI:** 10.3390/healthcare10020392

**Published:** 2022-02-18

**Authors:** Mark Vink, Friso Vink-Niese

**Affiliations:** 1 Independent Researcher, 1096 HZ Amsterdam, The Netherlands; 2 Independent Researcher, 49032 Osnabrück, Germany; frisovinkniese@gmail.com

**Keywords:** CFS, chronic fatigue syndrome, eminence-based medicine, evidence-based medicine, insurance medicine, long COVID, ME, occupational medicine, post-infectious disease, recovery behaviour

## Abstract

For the last few decades, medical guidelines have recommended treating patients with myalgic encephalomyelitis/chronic fatigue syndrome (ME/CFS) with graded exercise therapy (GET) and cognitive behavioural therapy (CBT). Moreover, doctors have questioned the recovery behaviour of these patients and stimulated them to follow these treatments so that they would be able to go back to work. In this article, we reviewed trials of GET and CBT for ME/CFS that reported on work status before and after treatment to answer the question of whether doctors should continue to question the recovery behaviour of patients with ME/CFS. Our review shows that more patients are unable to work after treatment than before treatment with CBT and GET. It also highlights the fact that both treatments are unsafe for patients with ME/CFS. Therefore, questioning the recovery behaviour of patients with ME/CFS is pointless. This confirms the conclusion from the British National Institute for Health and Care Excellence (NICE), which has recently published its updated ME/CFS guideline and concluded that CBT and GET are not effective and do not lead to recovery. Studies on CBT and GET for long COVID have not yet been published. However, this review offers no support for their use in improving the recovery of patients with an ME/CFS-like illness after infection with COVID-19, nor does it lend any support to the practice of questioning the recovery behaviour of these patients.

## 1. Introduction

Myalgic encephalomyelitis (ME), also known as chronic fatigue syndrome (CFS) or ME/CFS, is a seriously debilitating chronic multisystem disease, and not a psychiatric or psychosomatic one, according to the prestigious American Institute of Medicine (IOM), now known as the National Academy of Medicine [1], and the Dutch Health Council [2]. ME/CFS is a disease of unclear origins which often follows a viral or infectious illness [3,4]. It is characterised by delayed muscle recovery after trivial exertion [5], or exertion intolerance, which is currently often referred to as post-exertional malaise (PEM) [1]. This is an unusual, debilitating response to a low level of physical or mental exertion which previously did not cause any problems, and which substantially limits the functioning and quality of life of patients [1,2]. Other common symptoms include muscle pain, loss of muscle power, cognitive problems, reversal of the sleep rhythm and other sleep disturbances, dizziness, tinnitus, new-onset severe headaches, hypersensitivity to light and/or sound, and orthostatic intolerance [6]. Mild disease is defined by an approximate 50% reduction in functioning compared to pre-illness [7]. ME/CFS is associated with marked disability, reduced participation in social activities, long-term sickness absence, social isolation, and unemployment [8]. The American Centers for Disease Control and Prevention (CDC) reported that as many as 75% of patients (mean illness duration 14.3 years) were not working in a multicentre assessment study (n = 471) due to their ME/CFS [9], and less than a third of patients are estimated to resume employment within three years after diagnosis, as found by a systematic review [10]. Men, people in older age groups, and those who have been ill for longer periods of time are more likely to have ceased employment due to their illness [11].

However, most doctors think that ME/CFS is a psychosomatic illness. This view has been shaped by Surawy’s et al. cognitive behavioural (CB) model from 1995 and a similar model by Vercoulen et al. from 1998. According to these models, patients’ illness beliefs, coping strategies, and other behavioural factors are key factors in both the onset and perpetuation of the condition [12,13]. Patients avoid exercise to reduce their symptoms, but as a result, they become deconditioned. This results in chronic disability and the false belief that one has an ongoing, incurable illness. Cognitive behavioural therapy (CBT) tackles the illness-related cognitions and behaviours that work to maintain and perpetuate symptoms. Graded exercise therapy (GET) is used to improve the levels of physical activity and conditioning of the patient. On top of that, the graded exposure tackles the view that exercise and overexertion are feared stimuli. Ultimately, the aim of CBT and GET for ME/CFS is recovery from symptoms [14]. This has led to the predominant recommendation of CBT and GET as effective treatments. However, reviews by Song and Jason in 2005 [15] and Sunnquist and Jason in 2018 [16] showed that, if patients are selected who fulfil more stringent case definitions, so that ME/CFS patients are selected instead of those with a fatiguing psychiatric illness, then they do not possess the behavioural characteristics targeted by the CB model. Also, Geraghty et al. [17] concluded that “the CBM [CB model] is not fit for purpose, as it poorly reflects the accounts given by patients and it ignores the wealth of evidence showing biological, immune and neurological dysfunction in ME/CFS”. As noted by the developers of the model themselves [12], “the observations [upon which the model is based] have been made during treatment [of 100 patients] and require confirmation by objective measurement techniques”. Although this was written in 1995, this objective evidence has so far not been presented.

According to an influential systematic review by Cairns and Hotopf [10], only 5% of adult ME/CFS patients recover spontaneously, and patients should not be medically retired until they have been treated with CBT and GET first. Many CBT and GET studies have been published in recent years, partly as a result of this advice.

During a lecture on chronic fatigue complaints after infections [18], psychologist professor Knoop, who is one of the leading proponents of CBT for ME/CFS, gave a presentation on ME/CFS in which, as in some of his publications [19,20], he indicated that CBT is an effective therapy. The IOM, on the other hand, concluded that there is no effective therapy for this disease [1].

Until recently, Cochrane reviews and medical guidelines in many countries have recommended both treatments as effective treatments. There are several anecdotal stories from patients on the internet who have been ‘forced’ by medical professionals to undergo these treatments as a requirement to be eligible for illness benefits. This is, in part, based on the aforementioned review by Cairns and Hotopf [10], which recommended that “medical retirement should be postponed until a trial of such treatment [CBT and GET] has been given”.

If patients want to be eligible for illness benefits, then in many Western countries, there are also obligations for patients. For example, in the Netherlands, insured people have several obligations to prevent entitlement to benefits and to increase the possibilities for returning to work according to the Dutch Work and Income Capacity Act (WIA) [21]. According to the WIA, one of the obligations is that an insured person should be “treated medically or follow instructions from a doctor… and not impede his healing”. An insured person is also obliged to “undergo adequate treatment for his illness or defect by general medical standards”.

One of the guidelines that has been updated and changed in recent years is the Dutch ME/CFS guideline. The Dutch Health Council concluded that CBT and GET cannot be considered adequate medical treatments for ME/CFS [2]. A number of reviews have confirmed this conclusion and have highlighted the fact that CBT and GET do not lead to objective improvement and that the claim that they lead to recovery in around 20% of patients is incorrect [11,22,23,24,25]. Geraghty et al. [17] concluded that “there is little scientific credibility in the claim that psycho-behavioural therapies are a primary treatment for this illness”. However, the president of the Dutch Association of Insurance Physicians said in an interview in the *Medical Journal of the Dutch Medical Association* [26], that he does not agree that “if [ME/CFS] patients do not want to be treated with CBT and GET, then we can no longer dismiss this as ‘inadequate recovery behaviour’. Insurance doctors from the field of work and health were not involved at all in the advice of the Health Council: how shameless [een gotspe in Dutch]. We believe that we should be able to question the recovery behaviour of ME/CFS patients because we want to improve their state of health and because insurance also gives obligations in addition to rights. It would be a shame if patients refrain from potentially effective treatments and are permanently incapacitated for work. We therefore adhere to the existing 2013 ME/CFS guideline for insurance doctors”.

The scientific literature often uses the term ME/CFS; therefore, we will do the same in this article.

### Questions

We conducted literature research to determine whether it is useful for occupational and insurance physicians to question the recovery behaviour of patients with ME/CFS and stimulate them to be treated with CBT and/or GET, by answering the following questions:

1. What is the effect of CBT and/or GET on work status in ME/CFS post-treatment?
2. What percentage of patients return to full- or part-time work?
3. Are both treatments safe?

## 2. Methods

A literature review was conducted by searching the databases PubMed, Medline, the Cochrane Database of Clinical Trials, Academia, and Google Scholar for articles on the effectiveness of CBT and GET from January 1980 to December 2021. The search string (CFS OR chronic fatigue syndrome OR ME OR myalgic encephalomyelitis) AND (cognitive behavioural therapy OR CBT OR graded exercise therapy) AND (randomized controlled trial OR controlled clinical trial OR clinical trial OR evaluation study) was used. Further inclusion criteria were:

*Population*—adult patients aged 18 or older who had ME/CFS according to the study that had selected them (regardless of the criteria used).

*Outcomes*—only studies that reported on work status before the start of therapy and after finishing it were selected for our study.

*Design*—only randomized and controlled clinical trials, or evaluation studies were eligible for inclusion in our review.

*Language*—only studies written in English, German, or Dutch were included.

*Publication*—clinical studies were only included if they concerned peer-reviewed journal publications.

In addition, we also studied the references of the articles we found to try to find studies that did not appear in our searches.

### Exclusion Criteria

Studies without a control group were excluded, apart from evaluation studies. Studies with a badly designed control group, for example a ‘no treatment’ control group or a ‘waiting list’ control group, were not excluded.

A review of homeopathy studies by the Australian National Health and Medical Research Council (NHMRC), used a minimum number of 150 participants in randomised controlled trials because, according to the NHMRC, the results may be distorted in studies with a smaller number of participants [27]. We not only mention this review by the NHMRC because of the distortion of results if the number of participants in a study is small, but also because it was an integral part of the recent advice of the EASAC Homeopathy Working Group—The European Academies Science Advisory Council—to the EU on homeopathy [28]. Furthermore, one of its members was one of the world’s leading CBT proponents for ME/CFS. We therefore chose to use the same minimum number of participants (150, evenly distributed across the therapy and control group) for studies to be included in this review. As a result, randomised controlled trials with 149 participants or fewer were excluded from this review. We could also have chosen a minimum number of 100 participants, as this is the minimum number of participants for a Phase II trial [29]. However, even if we had done that, then the outcome of this review would not have been affected because the only study that would then also have been included in this review is the study by Jason et al. [30] of 114 participants, divided over 4 groups (CBT, cognitive therapy, aerobic activity, and a relaxation control group). The authors of this study concluded that there were no statistically significant differences in work status between the treatment and control groups after treatment. Moreover, the study by Huibers et al. (n = 151) [31], which is included in this review, noted the following: “To detect a clinically significant difference of 6 or more on the CIS at a two-sided significance level of 5% and a power of 80% we would need 63 patients per group. We extended the sample size to 75 patients per group to take into account potential withdrawal from the trial”. Ridsdale et al. [32], which is also included in this review, concluded that they would need “approximately 130 patients (65 per group)” to achieve something similar. White et al. [33], which is another study included in this review, concluded that, for “a two-sided test with 5% significance level and 90% power we calculated that the number of participants needed to compare SMC with APT was 135”. They “increased group size to 150 per group to allow for 10% dropout”. These studies did not mention what the estimated effect size (e.g., mean difference between the control group and treatment group) was in these power calculations. White et al. [33], the only study with a protocol, did not mention this in their protocol either [34].

## 3. Results

### 3.1. Selection of Articles

A total of 43 CBT and/or GET studies reported on the work status of participants. Of those, 27 studies were excluded because they only reported on work status before the initiation of therapy and not after the completion of it. Of the 16 remaining studies, 6 were excluded because they were studies with fewer than 150 participants; 3 of them were also non-randomized. As shown in Table 1, two of the remaining ten studies are studies in patients with chronic fatigue in which 43% and 28%, respectively, had ME/CFS [31,32]. Three studies were randomised, controlled trials involving a total of 1072 ME/CFS patients [33,35,36]. The remaining five studies are evaluation studies, involving a total of 2879 ME/CFS patients [37,38,39,40,41]. One of these five studies evaluated the efficacy of CBT in the Netherlands [37]. Another study evaluated the efficacy of 12 months of treatment with GET in the sports medical department of a Dutch hospital [38]. The other three were evaluations of CBT and GET in a London CFS clinic and in the official English and Belgian CFS clinics, respectively, carried out by proponents of these therapies for ME/CFS [39,40,41].

### 3.2. Drop-Outs

In Stevelink et al. [41], the dropout rate was 38%. Adamson et al. [43], who analysed the efficacy of CBT using data from the same clinic as Stevelink et al., noted that “those that dropped out reporting lower physical functioning scores with a mean difference of -7.38” than those who did not drop out. This confirms the conclusion by Lilienfeld et al. [44] that patients who drop out before the end of a study are often patients who do not benefit from a therapy or who deteriorate because of it, which can give the wrong impression about the effectiveness of a therapy.

The average drop-out rate in the clinical trials varied widely. In White et al. [33], it was 10.5% after CBT and 6.3% after GET. Compared to Prins et al. [36], in which it was 40.9% after CBT and 23.1% in the control group (no therapy), that was extremely low. However, in the same study by White et al., the Step Test results were missing in 29.8% (CBT) and 33.8% (GET). In Huibers et al. [31] and O’Dowd et al. [35], there was also a substantial difference in dropout rate between CBT (33% and 28.9% respectively) and no therapy (9.3% and 13.7%).

Van Berkel et al. [38] reported dropout rates over the course of 12 months of treatment with GET of 33%, 59%, and 72% after 6, 9, and 12 months, respectively. However, 64 patients who had dropped out of treatment before the three-month assessment, and who had not just started treatment, had not been referred to another specialty, or had not recovered, were excluded from the analysis. Yet, if one wishes to determine the effectiveness of a treatment and how well it is tolerated, then one should not exclude such patients. If they had been included, then the dropout rate after 6, 9, and 12 months would have been 55%, 73%, and 80%, respectively.

It is difficult to say what might explain the substantial variation in dropout rates because the studies used different outcome measures, and only the study by White et al. published a study protocol [34]. If we compare that study with the one by Prins et al. [36], then the latter did not provide sociodemographic or health-related background variables, contrary to the PACE trial by White et al. [33]. Prins et al. used the CIS fatigue scale, whereas White et al. used the Chalder fatigue scale. At baseline, patients in the CBT group in Prins et al. worked on average 16.3 h in 12 days. The PACE trial reported the percentage of patients on illness, disability, and other benefits, but not how many hours patients worked. The only notable difference between the two studies is that the patients in the CBT study by Prins et al. had been ill longer (4.9 years) than the patients in the PACE trial (36 months for CBT, and 35 months for GET). This might be one of the reasons for the difference in dropout rates, as an extensive review found that the prognosis in terms of returning to work is poor if patients have been on long-term sick leave for more than two to three years [11].

It is also impossible to tell if the substantial variation in dropout rates was caused by the fact that some studies might have included patients with missing data in their dropout rate, whereas others might not have done that, choosing to report those numbers separately.

These differences in dropout rates and the high dropout rates might have artificially inflated the efficacy of treatments under investigation.

### 3.3. Work Status

The main results of the 9 studies are summarised in Table 1. Studies by O’Dowd et al. [35] and Prins et al. [36], with a follow-up ranging from 6–14 months, found no statistically significant differences in work status between the treatment and control groups. More patients were back to work without treatment at 4 and 12 months, compared to patients who had been treated with CBT by their general practitioner in the study by Huibers et al. [31].

In the PACE trial (n = 641) by White et al. [33], the percentage of lost employment after CBT remained the same (84%) and increased from 83% to 86% after GET. Unemployment benefits increased from 10% to 13% (CBT) and from 14% to 20% (GET); disability benefits increased from 32% to 38% (CBT) and from 31% to 36% (GET); and private disability benefits increased from 6% to 12% (CBT) and from 8% to 16% (GET) [45].

The evaluation studies [37,38,39,40,41] found the following: In the Netherlands, patients worked 5 h per week less after CBT than before the start of it, and the percentage of patients who were working decreased from 41% to 31% [26]. Van Berkel et al. found that GET had no effect on work status at 3 months and 12 months. However, as so many patients dropped out, then it seems reasonable to assume that treatment, in reality, might have had a negative effect on the number of hours they were working. In the Belgian CFS clinics (n = 655), the percentage of people who were working decreased from 18.3% to 14.9% after treatment with CBT and GET, and the percentage of patients who were receiving incapacity benefits increased from 54% to 57% [40]. In the British CFS clinics (n = 952), the percentage of ME/CFS patients who could work decreased from 30% to 18% after treatment with CBT and GET [39].

There were several issues with the assessment of the employment status of 508 patients who attended a London outpatient CFS clinic before and after treatment. According to the article by Stevelink et al. [41], patients were diagnosed with CFS according to the NICE 2007 criteria although the reference is to the Oxford criteria. PEM, the main characteristic of the disease, is not required for diagnosis according to these criteria. However, according to a study by the same authors using the same group of patients from 2019 [46], “a CFS diagnosis was confirmed by a clinician using the Oxford criteria”.

The authors concluded that “the 9% of patients returning to work in this study is heartening and suggests that people can recover, as previously found in both randomized controlled trials and routine clinical practice. These findings suggest that there is some room for optimism in terms of CFS improving to such an extent that a return to employment is possible for a small, but significant number of patients”. However, only 316 (62%) patients provided follow-up data. Even though 9% had returned to work at follow-up, 6% were unable to continue to work at follow-up after 285 days. This means that, compared to baseline, only 3% returned to work, not 9%. According to the authors, “unhelpful beliefs such as fear of activity and exercise and concerns about causing damage, combined with all or nothing behaviour and behavioural avoidance, were associated with not working and are specifically targeted in CBT and, to some extent, GET”. However, the authors did not mention that, after a more than adequate number of treatment sessions, on average 16.5 treatment sessions, the scores for fear-avoidance, catastrophizing, damage, embarrassment avoidance, symptom focusing, all or nothing behaviour, and avoidance/resting behaviour did not change. This means that, even though these symptoms are “specifically targeted in CBT and, to some extent, GET”, their own study shows that neither treatment had any effect on these problems. Also, the fatigue severity score remained the same (26), and the physical functioning score changed only marginally (from 47.5 to 50). Anxiety caseness remained the same (46%), so CBT did not affect anxiety either. As far as depression was concerned, depression caseness improved from 30% to 28%. So, it might well be that these 2% made up most of the net 3% increase in patients who were able to work at follow-up. On top of that, the study included people who were already well enough to work at baseline yet were unemployed. These patients should have been excluded from this study because, in their cases, returning to work would not reflect the efficacy of the treatment. That this is not an arbitrary problem is highlighted by the fact that 23% (53/229) of the patients who were classified as not working at baseline were already well enough to work before they had received any treatment. This is reminiscent of the PACE trial [33] by the corresponding author of Stevelink et al. [41], where 13.3% of patients already fulfilled recovery criteria at baseline before they had received any treatment, as found by a reanalysis of that study [23].

Also at baseline, 34% of patients had been ill for less than 2 years, and this had increased to 39% at follow-up. The prognosis for recovery and substantial improvement that enables a return to work is poor if patients have been off work for 2 to 3 years according to a review [11]. These figures suggest that patients who had been ill for longer were less likely to provide follow-up data.

## 4. Safety

The Do No Harm principle is a basic principle of medicine [47]. One can therefore only strongly stimulate or force patients to be treated with a certain treatment if that treatment is safe. However, most of the clinical trials in this review did not report on safety, as can be seen in Table 2. Huibers et al. [31] reported no adverse events after CBT by GPs. The PACE trial by White et al. [33] reported that CBT and GET are safe, but they assessed safety in the following manner: “Safety was assessed primarily by recording all serious adverse events, including serious adverse reactions to trial treatments,” and “adverse events were considered serious when they involved death, hospital admission, increased severe and persistent disability, self-harm, were life-threatening, or required an intervention to prevent one of these”. Patient surveys on the other hand, have shown over the last 20 years that both therapies often exacerbate ME/CFS symptoms, cause relapses, and lead to (severe) health deterioration [48,49,50]. In other words, patients do not complain about the things that were used by the PACE trial to assess the safety of CBT and GET.

As mentioned earlier, there was a very high dropout rate (80%) in the sports medical department of a Dutch hospital where patients were treated for 12 months with GET. At baseline, 66.7% of patients in this study were working or studying, on average 28.4 h per week. This suggests that many patients in the study were only very mildly affected. Consequently, it seems reasonable to assume that GET was not very well tolerated or safe for these patients either.

The Oxford Brookes University [51] published a study (n = 2274) in 2019 on the safety of CBT and GET as part of the review of the National ME/CFS guidelines in England. Worsening of symptoms after treatment was reported by 58.3% (CBT, which incorporates an element of GET in ME/CFS) and by 81.1% (GET). In addition, the percentage of patients who were bedridden and dependent on help from others due to severe ME/CFS increased from 12.6% to 26.6% after treatment (CBT, which incorporates an element of exercise therapy in ME/CFS) and from 12.9% to 35.3% (GET).

## 5. Discussion

Up to 75% of ME/CFS patients are too ill to work [1]. Many patients from around the world have been treated with CBT and GET as a requirement for being eligible for illness benefits and medical retirement because of an influential systematic review from 2005 that concluded that medical retirement should be postponed until patients have tried CBT and GET [10]. In this review, we analysed the work outcomes of 3721 patients who were treated with CBT and/or GET to determine whether it makes sense to question the recovery behaviour of ME/CFS patients, as the president of the Dutch Society for Insurance Physicians said that insurance doctors should do that to reduce the number of ME/CFS patients who are unable to work and are receiving disability benefits [26]. One of the studies in our analysis was Prins et al. (n = 278) [36]. According to the actometer results of this study—which were not published until 9 years later—CBT does not lead to objective improvement [20]. In addition, after 8 and 14 months of follow-up, there was no statistically significant difference in the number of hours worked between those who received CBT and those who received no treatment.

In the PACE trial (n = 641) by White et al. [33], the number of patients receiving incapacity benefits increased after treatment with CBT and GET [45]. Analysis of the use of CBT in the Netherlands [26], as well as evaluation of the efficacy of CBT and GET in the official English and Belgian CFS clinics, showed that the number of people who could work decreased after treatment with CBT and GET [39,40]. Moreover, the Belgian evaluation, like the PACE trial, showed that neither therapy leads to objective fitness improvement in patients. This is not surprising when one considers that the sickness *response*, which is partly responsible for the manifestation of exertion intolerance or post-exertional malaise, the main characteristic of the disease, is part of the underlying mechanism that indicates a complex disruption with, among other things, disorders in aerobic cellular energy production [1,6]. Moreover, a large number of studies have provided objective evidence for the exertional intolerance, delayed muscle recovery, and other physical abnormalities in ME/CFS following exercise, which are not seen in healthy (sedentary) controls [52,53,54,55,56,57,58,59,60,61].

Unfortunately, the medical profession has not followed this research up to try to determine the underlying physiological reasons why patients respond abnormally to exercise and exertion. Instead, we have been spending the majority of our research money on doing yet another CBT and/or GET study. Consequently, we can only speculate what might cause the exertion intolerance in ME/CFS. One possibility is that ME/CFS is caused by a chronic infection. A study by Shor [62] found that a substantial subgroup of ME/CFS patients in a Lyme endemic environment actually had a perpetuation of symptoms driven by a persistent but seronegative infection with Borrelia burgdorferi. Treatment with appropriately directed antimicrobials improved the outcomes.

According to a number of reviews, another possibility is that persistent pathogens or reactivation of latent pathogens (for example herpes viruses) drive chronic symptoms in ME/CFS by interfering with host metabolism, gene expression, and immunity. Patients can have similar clusters of inflammatory symptoms because different human pathogens have evolved similar survival mechanisms to disable the host immune response and host metabolic pathways [3,63]. The exertion intolerance and other ME/CFS symptoms might also be caused by virally induced autoimmunity [62,63]. Interestingly enough, similar mechanisms are also thought to play a part in the development of long COVID [64,65,66].

This raises the question of whether it is ethical for medical professionals to try to convince patients that there is no underlying physical illness and that the patients are only suffering from abnormal cognitions and deconditioning. The therapist manual of the GETSET trial for example [67] states that “participants are encouraged to see symptoms as temporary and reversible, as a result of their current relative physical inactivity, and not as signs of progressive pathology”. According to the FINE trial [68], which treated the more severely affected patients at home [69], ME/CFS “is a good news diagnosis” and “exercise can reverse the condition” because “there is no disease”. It also raises the question as to whether it is ethical to treat patients with GET and force them to go over their limits, as objective evidence to support the notion that there is no underlying disease has never been presented. Patients, on the other hand, are convinced that they have a physical disease. Research findings over the last few decades have provided solid scientific insights that contradict the CB model and confirm that ME/CFS is a seriously debilitating, chronic, multisystem disease with many organic dysregulations, and not a psychiatric or psychosomatic one, as concluded by the American Institute of Medicine [1].

Stevelink et al. [41] concluded that “further research efforts should be directed into exploring the effectiveness of interventions to help CFS patients maintain their job or re-enter the work force when symptoms subside”. Our review shows that CBT and GET are not the interventions patients, employers, and society need to achieve that.

Continuing to use these therapies is an irresponsible choice because of the great risk of the aggravation of complaints and the cause of serious, and possibly even permanent, relapses.

Our conclusions confirm the conclusions of the British National Institute for Health and Care Excellence (NICE), which recently published their updated ME/CFS guideline, in which they concluded that CBT and GET are not effective and do not lead to recovery [70] Furthermore, GET is harmful and should therefore not be used. Moreover, CBT as defined by Beck, should only be offered as a supplementary treatment if patients suffer from comorbid anxiety or depression.

According to several reviews, a substantial number of patients are developing long COVID after an infection with COVID-19 [71,72,73,74]. According to NICE [75], this term includes ongoing symptomatic COVID-19, from 4 to 12 weeks post-infection, and post-COVID-19 syndrome, beyond 12 weeks post-infection. As noted by, for example, Dr. Anthony Fauci, Director of the National Institute of Allergy and Infectious Diseases in the United States [76], the symptoms experienced by those suffering from long COVID beyond 12 weeks, are very similar to those experienced by ME/CFS patients. Data from a large, international, web-based patient survey indicate that 75% of respondents with long COVID reported suffering from post-exertional symptom exacerbation, the main characteristic of ME/CFS [77]. Studies on CBT and GET for long COVID have not yet been published. However, this review offers no support for their use for improving the recovery of patients with an ME/CFS-like illness post-infection with COVID-19 or to question their recovery behaviour.

### Strengths and Weaknesses of This Review

A weakness of this review is that most clinical trials in this review did not report on the safety of the therapies.

Strengths of the review are:

- inclusion of work outcome analysis in a very large number of patients (3721) before and after treatment with CBT and GET;
- inclusion of the evaluation reports of the official Belgian and English CFS clinics by advocates of CBT and GET with >1600 patients not specially selected for a clinical trial, so that it becomes clear what the effectiveness of both therapies in daily life is;
- inclusion of the research report of an English university (n = 2274) on the safety of both therapies in the context of the revision of the National ME/CFS guideline in England.

## 6. Conclusions

Our review shows that more patients are unable to work after treatment with CBT and GET than before treatment with it. Moreover, there is also a high risk that CBT and GET lead to the worsening of complaints and cause serious relapses, from which patients might not recover. Therefore, questioning the recovery behaviour of patients with ME/CFS is not only pointless, but it also goes against the Do No Harm principle of medicine. The outcome of this study supports the conclusion of the Dutch Health Council that CBT and GET do not constitute adequate treatments according to general medical standards. It also supports the conclusion of the British NICE Institute in its recently published, updated guideline, that neither treatment is effective, nor do they lead to recovery. CBT and GET studies for long COVID have not been published, but our review does not lend support for questioning the recovery behaviour of patients with an ME/CFS-like illness after COVID-19, either.

## Figures and Tables

**Table 1:** Work status before and after treatment with CBT and/or GET.

Study	Intervention	N	Criteria	FU Length	Control Group	Work Outcome	Dropouts
Collin and Crawley [39]	CBT and GET evaluation in 11 English CFS clinics	952	NICE	1 yr	Evaluation study	After therapy: 47.2% unchanged working status; 18.0% worked again or longer; 30.0% stopped working or worked less because of CFS	Response rate: 46.2%
Huibers et al. [31]	CBT by general practitioners	151 (fatigue; 43% CFS)	Fukuda	12 mo	No treatment	After 4 mo 50% (CBT) and 61% (NT) and after 12 mo 59% (CBT) and 65% (NT) were back at work.	33% CBT, 9.3% NT
Koolhaas et al. [37]	Evaluation of CBT in The Netherlands	100	Fukuda	Evaluation study	Evaluation study	41% were employed before and 31% after CBT; patients who worked, worked 5 h less after CBT	Response rate: 100%
O’Dowd et al. [35]	GrCBT with graded activity	153	Fukuda	12 mo	No treatment	The authors concluded that group CBT did not significantly improve employment status.	No cognitive test data: 28.9% CBT, 13.7% NT
Prins et al. [36]	CBT vs. Guided Support	278	Oxford	14 mo	No treatment	No statistically significant difference in the number of hours worked after 8 (p = 0.3362) and 14 mo (p = 0.1134) between CBT and NT	40.9% CBT and 23.1% NT
Ridsdale et al. [32]	CBT vs. counseling	160 (fatigue; 28% CFS)	Fukuda	6 mo	Counselling	Number of sick days decreased by 4.3% (counselling) vs. increased by 6.6% (CBT)	36% counselling and 31% CBT
Stevelink et al. [41]	CBT (285), GET (28), APT (2), CBT and GET (1)	508	Oxford	285 days	Evaluation study	On average 16.5 treatment sessions. Fatigue and physical functioning scores did not improve; 9% returned to work, 6% stopped working, net improvement 3%, depression caseness improved by 2%; 23% (53/229) of patients who were classed as not working at baseline, were already well enough to work before they had received any treatment.	38%
Stordeur et al. [40]	CBT and GET evaluation in Belgian CFS clinics	655	Fukuda	Evaluation study	Evaluation study	Work status decreased from 18.3% to 14.9%; percentage of incapacitated persons increased from 54% to 57%	28%
Van Berkel et al. [38]	GET evaluation in sports medical department of Dutch hospital	123	Fukuda	12 months	Evaluation study	Work status at 3 and 12 months did not change	33% (6 months) and 72% (12 months)
White et al. [33]	CBT vs. GET vs. APT	641	Oxford	52 wks	SMC (no treatment)	Lost working years remained 84% (CBT); increased from 83% to 86% (GET). Unemployment rates increased from 10% to 13% (CBT) and from 14% to 20% (GET); disability benefits increased from 32% to 38% (CBT) and from 31% to 36% (GET); private disability benefits increased from 6% to 12% (CBT) and from 8% to 16% (GET)	10.5% CBT, 6.3% GET. Missing step test data: 33.8% GET and 29.8% CBT

The working results of Ridsdale et al. [32] and White et al. [33] were published in their economic evaluation (Chisholm et al.) [42] and cost effectiveness analysis (McCrone et al.) [43], respectively. APT: adaptive pacing therapy; CBT: Cognitive behavioural therapy, often abbreviated to behavioural therapy; CT: cognitive therapy; FU: follow-up; GrCBT: Group CBT; GET: Graded exercise therapy; mo: months; NT: no treatment (no therapy); SMC: specialist medical care; vs.: versus; WL: waiting list; wks: weeks.

**Table 2:** Reporting of adverse outcomes.

Study	Adverse Outcomes Reported	Intervention	Adverse Events
Collin and Crawley [39]	Yes	CBT and GET	Overall change in health: 20.1% felt worse at 1-year and 30.6% at 5-year follow-up
Huibers et al. [31]	Yes	CBT by GPs	None
Koolhaas et al. [37]	Yes	CBT	38% negatively affected by CBT
O’Dowd et al. [35]	No	Group CBT with Graded Activity	Not reported
Prins et al. [36]	No	CBT vs. guided support	Not reported
Ridsdale et al. [32]	No	CBT vs. counseling	Not reported
Stevelink et al. [41]	No	CBT vs. GET vs. APT vs. CBT and GET	Not reported
Stordeur et al. [40]	No	CBT and GET	Not reported
Van Berkel et al. [38]	Yes	GET	Increase in tiredness: 13.7% (3 months) and 11.5% (12 months)
White et al. [33]	Yes	CBT vs. GET vs. APT	SAE: 1% APT, 2% CBT, 1% GET and 1% SMC

SAE: serious adverse reactions to trial treatments: “adverse events were considered serious (by White et al. [33]) when they involved death, hospital admission, increased severe and persistent disability, self-harm, were life-threatening, or required an intervention to prevent one of these”.
